# Gastrointestinal stem cell up-to-date

**Published:** 2015

**Authors:** V Pirvulet

**Affiliations:** * Department of Cell Biology and Histology, ”Carol Davila” University of Medicine and Pharmacy, Bucharest, Romania

**Keywords:** gastrointestinal stem cell, intestinal stem cell,, cancer stem cell, stem cell up-to-date

## Abstract

Cellular and tissue regeneration in the gastrointestinal tract depends on stem cells with properties of self-renewal, clonogenicity, and multipotency. Progress in stem cell research and the identification of potential gastric, intestinal, colonic stem cells new markers and the signaling pathways provide hope for the use of stem cells in regenerative medicine and treatments for disease. This review provides an overview of the different types of stem cells, focusing on tissue-restricted adult stem cells.

## Introduction

Homeostasis of the normal adult gastrointestinal epithelium is maintained by rapid and continuous replacement of differentiated cells by proliferation of undifferentiated epithelial cells located within the crypts (intestinal epithelium) or glands (gastric epithelium) and further differentiation of their progeny during migration away from the zone of replication. 

The epithelium of the whole intestinal tract is a tissue with rapid turn-over. This renewal process involves the proliferation of the epithelial cells at the base of the crypt with subsequent migration of these cells along the crypt-villus axis. An intestinal stem cell (ISC) is defined by three properties: the ability to maintain itself throughout long periods of time (self-renewal), clonogenicity and the potential to generate differentiated cell types of the intestinal epithelium - enterocytes, goblet cells, enteroendocrine cells, and Paneth cells (multipotency). 

Intestinal stem cells divide asymmetrically, giving rise to an identical stem cell and a committed progeny. The advances in our understanding of stem cells in the gastrointestinal tract include the identification of molecular markers of stem, stem-like and early progenitor cells. 

This review highlights recent advances on gastrointestinal stem cells and their involvement in the regenerative processes of the digestive tract in health and disease.

**Gastric Epithelial Stem Cells**

The mammalian adult gastric epithelium self-renews continually through the activity of stem cells located in the isthmus of individual gland units. Although the gastric epithelial stem cells have been localized, little is known about their molecular biology. Researchers have begun to identify the signaling pathways and events that take place during embryonic development that eventually establish the adult stem cells to maintain the specific features and functions of the stomach mucosa. [**1**]. Recent reports describe the use of inducible Cre recombinase activity to label candidate stem cells and their progeny in the distal stomach (the antrum and pylorus). No such labelling of the epithelial stem cells lineage has been reported in the gastric body (corpus) [**1**]. Recently identified stomach stem cells are restricted to the antral-pyloric segment and a small area of the corpus near the squamocolumnar junction; they lie close to the gland base, and like the intestinal crypt base columnar cells [**2**], they express the cell surface marker Lgr5 [**3**].

Among stem cells in the alimentary canal, those of the adult corpus are unique as they lie close to the lumen and increase the proliferation following the loss of a single mature progeny lineage, the acid-secreting parietal cell. They are also unique as they neither depend on Wnt signaling pathway nor express the surface marker Lgr5 [**1**].

The corpus stem cell gives rise to four functionally distinct cell lineages: parietal, surface mucous (pit), zymogenic, and enteroendocrine. The zymogenic lineage and its microenvironment are particularly amenable to developmental analysis, because zymogenic lineage differentiation proceeds in an orderly spatiotemporal pattern in which the degree of maturation correlates with the distance migrated from the stem cell. The zymogenic progenitor cell, known as the mucous neck cell (NC), migrates through the neck, or middle, of the gastric unit towards the gastric unit base. These progenitors are long-lived (~ 14 days) and express markers that distinguish them from both the stem cell and the mature zymogenic cells (ZCs) to which they will ultimately give rise in the base [**4**]. The zymogenic cells are remarkably plastic. Normally, they are postmitotic and long-lived. In the face of metaplasia-inducing injury (e.g., experimentally induced loss of XBP1 or, in certain patients, infection with the pathogen *Helicobacter pylori*), they can re-enter the cell cycle and then regain stem cell properties, fuelling the differentiation of other gastric lineages. In other words, they are an example of a naturally occurring, induced multipotent stem cell. The genes involved in these transitions may be at the root of gastric cancer [**1**]. 

Because the pathogenesis of gastric adenocarcinoma has been associated with abnormal patterns of gastric differentiation and with chronic tissue injury, there has been much research on the response of stomach epithelial stem cells to inflammation. The chronic inflammation, as induced by infection with *Helicobacter pylori*, affects the differentiation and promotes metaplasia [**1**]. 

In particular, Wnt–β-catenin signaling is essential for the proliferation of the intestinal crypt cells [**5**], but its functional requirement in most gastric stem cells is unclear. Wnt activation in Lgr5+ intestinal or stomach antral stem cells induces adenomas [**3**,**6**]. Notch and Wnt signaling pathways seem to cooperate in intestinal tumorigenesis. Notch signaling occurs in the mouse stomach epithelium during development and becomes restricted mainly to the isthmus in adult glands. Recent studies revealed a requirement for Notch signaling in gastric epithelial homeostasis, affecting the proper balance between the self-renewal and differentiation that is essential to avoid tumours [**7**].

Recent evidence suggests that Notch regulates progenitor proliferation and secretory cell differentiation through a single intestine-restricted basic helix–loop–helix transcription factor, Math1. It is also unknown if the Notch pathway affects the gastric corpus progenitors, which are not strictly Wnt dependent, or promotes gastric tumorigenesis as it does in cooperation with Wnt in the intestine [**8**].

**Intestinal stem cells**

In 2007, Hans Clevers and his group found the Wnt target gene leucine-rich-repeat-containing G-protein coupled receptor 5 (Lgr5) to be expressed in cells at the crypt base [**2**]. Furthermore, irreversible labelling of these cells revealed that all 4 epithelial cell types (columnar cells, goblet cells, Paneth cells and neuroendocrine cells) arise from these cells. Since LGR5-positive cells are located at the crypt base, are pluripotent and also self-renewing, they concluded that LGR5 is a good marker for the intestinal stem cells [**2**]. 

Assuming a murine crypt population of around 250 cells, stem cells appear to comprise only 5% of this total population [**9**]. Various markers have been used to identify intestinal stem cells based in the main on the utilisation of mouse models; these include CD133, CD44, CD24, Bmi1 and Lgr5. Many of these identified markers have overlapping expression patterns and are often implicated in various aspects of the canonical Wnt signaling pathway, which is strongly associated with both normal intestinal stem cell function and colorectal carcinogenesis [**10**]. However, several of these markers, have problems with specificity and while overlaying stem cell populations they also mark other non-stem cells. CD24 exemplifies this issue; while having been shown to be a bona fide stem cell marker in one report, an apparently conflicting account also showed that CD24 is a marker of Paneth cells [**10**]. A careful appraisal of these papers showed that CD24 has variable expression levels; while CD24Low/ Mid marks the intestinal stem cell compartment, CD24High marks Paneth and enteroendocrine cells. Of all the markers described to date, Lgr5 has been shown to unequivocally and specifically mark the intestinal stem cell compartment as demonstrated through *in vitro* culture and *in vivo* lineage tracing studies [**2**]. Lgr5-positive cells are rapidly cycling and have been shown by using a variety of approaches to have a cell cycle time approximating to around 24 h [**11**]. Also, by the analysis of clonal population dynamics, it has been shown that in the physiological situation, there can exist only one equipotent but potentially heterogeneous stem cell population [**12**]. These data suggest that the whole intestinal stem compartment in the normal physiology is rapidly cycling but they do not address the possibility of plasticity during times of injury, that is, a cell acquiring stem-like characteristics. 

In 2009, Hans Clevers and his group found olfactomedin 4 (also known as OLFM4, hGC1 or GW112), a glycoprotein characteristic of the intestinal stem cells, to be a robust and specific marker for these LGR5 stem cells in the human intestine [**13**].

*In vivo*, two putative stem cell markers have been rigorously evaluated: leucine-rich repeat containing G-protein-coupled receptor (Lgr5) [**2**], and Bmi1 [**14**]. Others, including Dcamkl1 (double cortin and calcium/ calmodulin-dependent protein kinase-like-1), Musashi-1, a microtubule associated kinase, β1-integrin, phosphorylated PTEN, 14-3-3£, phosphorylated Akt, and sFRP-5 (secreted frizzled-related protein 5), a Wnt antagonist, have been proposed as intestinal stem cell markers based on their position in the crypt base, just above Paneth cells [**5**,**15**]. Other markers of the crypt base columnar cells population include olfactomedin 4 (Olfm4) and Achaete scute-like 2 (Ascl2) [**16**].

Gene signature evaluation of Lgr5 + stem cells has led to the identification of *Olfactomedin-4 (Olfm4*), a member of the bone morphogenetic protein (BMP) antagonist family expressed in murine small intestine but not in murine colon. However, in humans, Olfm4 is highly expressed in both colon and small intestine crypt base columnar cells as well as in a subset of cells within adenocarcinomas [**13**]. Unlike Ascl2, Olm4 is not detected in adenomas, suggesting it is not expressed under control of the Wnt pathway [**17**].

Prominin1 (also called CD133; a pentaspan transmembrane glycoprotein that localizes to membrane protrusions) is another marker that co-localizes with Lgr5+ cells but is also found in the proliferating progenitor cells [**18**]. Lgr5+ stem cells can be maintained in long-term culture and differentiated into crypt-villus like units, if grown in laminin-rich Matrigel in the presence of the Wnt agonist R-spondin 1, the Bmp antagonist Noggin, a Notch agonist peptide, epidermal growth factor and the Rho kinase inhibitor Y-27632 which inhibits embryonic stem cell anoikis. Thus, a single Lgr5+ stem cell can give rise to crypt-villus-like structures in the presence of the appropriate extracellular signals, which are derived from the underlying mesenchyme *in vivo*.

Utilizing a microenvironment consisting of an air-liquid interface 3D collagen gel to improve oxygenation, as well as myofibroblasts and stem cell niche signaling molecules, long-term culture of intestine and colon has been established [**16**].

Intact Wnt and Notch signaling, recognized as active in the intestinal stem cell niche, were confirmed to be required for the *in vitro* cultures. The presence of intestinal stem cells was confirmed by examining Lgr5 and Bmi1 expression. Stemness is principally not an intrinsic cell-defined property; instead, it appears to be determined by proximity to contextual cues from the stem cell niche. A spectrum of stem-cell competence exists with variable bias towards self-renewal or differentiation dependent on distance from a “sweet spot” in the crypt base [**19**].

Consistent with this spectrum, quiescent or reserve stem cell populations, and different secretory precursor cells that have ostensibly exited the niche, have the ability to reactivate stem cell potential at times of need and regenerate the crypt when damaged [**20**,**21**].

Intestinal stem cells are regulated by several cell signaling pathways, including the Wnt, Notch, BMP, PI3K pathway [**22**,**23**]. Defects in these pathways are known to be related to the development of intestinal cancer [**23**-**25**].

**Cancer stem cells**

It is now widely accepted that tumour maintenance is a function of a subset of stem-like or cancer stem cells (CSCs). The majority of the currently identified intestinal stem cell populations appear to be rapidly cycling. However, quiescent stem cell populations have been suggested to exist in both normal intestinal crypts and tumours. Cancerous cells have various strategies to evade toxicity from chemotherapy and radiotherapy, one of which is the homeostatic phenomenon of cellular quiescence. The relative contribution of quiescent and continuously dividing stem cell populations in maintaining both normal intestinal tissue and malignant colorectal tumours remains far from clear. Both populations appear to coexist in the intestine [**10**]. 

It was demonstrated that only a discrete sub-population of cells has tumour-initiating capacity. It still remains unclear if these are transformed “normal” stem cells that have undergone a malignant change and yet retain their “stem”-like characteristics or, alternatively, if they are differentiated malignant cells that have re-acquired stem-like characteristics [**26**].These two possibilities can both occur, and it is not known in which tumours or specific circumstances they appear. It is not necessarily to presume that the stem cell is the cell of origin of the tumour although this may be the case in the intestine [**6**].

The identification of CSCs has been dominated by the use of cell surface markers to isolate tumour cell sub-populations and also assessing their tumour-initiating capacity. Interestingly, many putative CSC markers not only appear to mark CSCs from disparate tissues but also appear to overlap significantly with the normal stem cell markers. For example, CD24 has been shown to not only mark stem cells in the normal intestine and lung but also the CSC populations in the colon, ovary and pancreas [**10**]. Although these findings suggest overlapping regulatory functions, the situation is far more complex. For example, CD24 marks normal mammary stem cells but in combination with the other cell surface markers, CD24-negative breast cancer cells are those with the greatest tumour-initiating potential. Further, although these cell surface markers can successfully isolate stem cell populations, the protein function may not be directly related to stem cell function. It has also been suggested that quiescent stem cells exist as a conditional reservoir that only becomes active after periods of injury where there is a loss of the rapidly cycling stem cell population. Quiescent CSCs have been isolated from melanoma, ovarian, breast and pancreatic tumours [**10**].

Lgr5-positive cells have been shown to be representative of the cell of origin of intestinal tumorigenesis and have tumour-initiating potential [**6**]. Moreover, CD133 and CD24 expression have also been shown to relate to the degree of differentiation and invasiveness of colorectal cancer [**27**]. The loss rather than the gain of the membranous expression of the CSC markers CD44, CD166 and EPCAM is associated with colorectal tumour progression [**28**]. As it has not yet been demonstrated that any one, or combination of CSC markers is capable of capturing the CSC sub-population throughout the development of a tumour, it remains possible that the sub-populations may be missed. Furthermore, the demonstration that murine intestinal cancers can arise from cells which are normally situated outside the crypt base [**29**] or from dedifferentiated cells [**30**] in an altered mucosal microenvironment, it means that there is a pressing need to expand our knowledge of the cell of origin of human tumours.

## Conclusions

The possible connection between the quiescent and the actively cycling nature of the intestinal stem cells needs to be further investigated. Furthermore, it is critical to understand the genetic elements that determine the fate of the stem cell and the basis according to which regeneration occurs in order to better understand stem cell plasticity and the contribution of the stem cell compartment to the malignant disease. 

The existence of quiescent colonic and rectal CSCs remains largely unexplored due to the current lack of a definitive marker. 

Identifying, characterising and developing novel targeting strategies against CSCs should not only increase the efficacy of adjuvant therapies but also enable the identification of patients at risk of disease recurrence through poor response to treatment.
